# Does School-Based Health Promotion Affect Physical Activity on Weekends? And, Does It Reach Those Students Most in Need of Health Promotion?

**DOI:** 10.1371/journal.pone.0137987

**Published:** 2015-10-21

**Authors:** Kerry A. Bastian, Katerina Maximova, Jonathan McGavock, Paul Veugelers

**Affiliations:** 1 Population Health Intervention Research Unit. School of Public Health, University of Alberta, Edmonton, Alberta, Canada; 2 School of Public Health, University of Alberta, Edmonton, Alberta, Canada; 3 Manitoba Institute of Child Health, Department of Pediatrics and Child Health, Faculty of Medicine, University of Manitoba, Winnipeg, Manitoba, Canada; University of St Andrews, UNITED KINGDOM

## Abstract

**Objective:**

To determine whether a school-based health promotion program affects children’s weekend physical activity and whether this effect varies according to socioeconomic-status.

**Methods:**

This was a quasi-experimental trial of school-based programs on physical activity levels implemented in disadvantaged neighborhoods in Alberta, Canada. In 2009 and 2011, 7 full days of pedometer data were collected from cross-sectional samples of grade 5 students (age 10–11 years) from 10 intervention schools in low-socioeconomic neighbourhoods and 20 comparison schools in middle-socioeconomic neighbourhoods. Multilevel models assessed differences in step-counts between intervention and comparison groups over-time by weight (objectively measured) and socioeconomic status subgroups.

**Results:**

In 2009, children from intervention schools were less active on weekends relative to comparison schools (9212 vs. 11186 steps/day p<0.01). Two years later, daily step-counts on weekend days among children in low socioeconomic intervention schools increased such that they approximated those of children from middle socioeconomic comparison schools (12148 vs. 12121 steps/day p = 0.96). The relative difference in steps between intervention and comparison schools on weekends reduced from -21.4% to 0.2% following the intervention. The normalization of weekend step counts was similar for normal weight (–21.4% to +2.0%) and overweight (-19.1 to +3.9%) children, and was balanced across socioeconomic subgroups.

**Conclusions:**

These data suggest that school-based health promotion is effective for reducing inequities in physical activity levels outside school hours. Investments in school-based health promotion lead to behavior modification beyond the school environment.

**Trial Registration:**

ClinicalTrials.gov NCT01914185

## Introduction

Physical activity is important for short- and long-term health outcomes in youth. Regular physical activity, particularly at moderate to vigorous intensity, reduces the risk of several chronic diseases and promotes mental health and well-being [1]. Though these benefits are widely recognized, the majority of children and youth in Canada [2] and elsewhere [3] do not engage in the recommended 60 minutes of moderate-to-vigorous physical activity daily. Further, a series of recent studies has demonstrated that children’s physical activity levels are lower on weekend days than on school days [4–7]. Physical activity levels, like smoking, poor diet and chronic diseases disproportionately affect children in socioeconomically disadvantaged households [8–12]. As physical activity habits of youth track into adulthood [13], social discrepancies in childhood may perpetuate inequalities in physical activity into adulthood and the next generation. Accordingly, interventions that reduce the inequity in physical activity among socioeconomically disadvantaged children may reduce future health inequalities.

Schools are an important setting for physical activity promotion targeting children. Given that children’s physical activity levels are particularly low after school hours and on weekends, fostering physical activity outside school hours has become a key component of many health promotion programs [14]. Though only recently has there been evidence for the effectiveness of school-based programs to improve children’s physical activity during these windows [15]. It has been suggested that children who are overweight or come from socioeconomically disadvantaged families may require additional support to become active outside of the school setting [9, 16, 17]. It has also been suggested that without this additional effort, these children may fall further behind in physical activity after school hours and on weekends. These hypotheses, however, have not been documented empirically in the literature. If physical activity related inequalities are in fact exacerbated outside of school among children already at risk of low physical activity, it would be especially important to ensure that these children are receiving benefits from school-based health promotion programs that are equal to or greater than those received by their more active and advantaged peers. To our knowledge, no experimental studies exist assessing the effectiveness of a school-based intervention to reduce physical activity inequities among children outside of the school setting.

To overcome these limitations, the aims of the current study were to determine 1) if physical activity related inequalities are exacerbated outside of school (i.e., weekend days and before or after school hours) among children already at risk of low physical activity, 2) if school-based health promotion programs reduce or increase physical activity related inequalities evident outside school, and 3) if the effects of school-based health promotion programs are modified by body weight status and socioeconomic status. We hypothesized inequalities in children’s physical activity levels would be exacerbated outside of school. We also hypothesized that compared to students receiving standard curriculum, students in schools implementing a comprehensive school health (CSH) program would display greater increases in daily physical activity outside of school, and that overweight and socioeconomically disadvantaged children would receive benefits from the intervention that were equal to or greater than their normal weight and more advantaged peers.

## Methods

### Study Design

The current study used a quasi-experimental pre-post design with a parallel non-equivalent comparison group to test the study hypothesis. The Alberta Project Promoting active Living and healthy Eating in Schools (APPLE Schools) began in January 2008 and lasted through June 2011, and was implemented school-wide. As such, all students attending a school participating in the APPLE Schools intervention received the intervention. The APPLE Schools intervention is committed to schools “in need of health promotion”, therefore schools were not randomly allocated to intervention and comparison groups. Rather, schools were considered for the intervention if they were located in socioeconomically disadvantaged neighbourhoods and the school principal was willing to support the intervention and research. Based on these criteria, an advisory panel representing 5 school jurisdictions identified 10 potential schools in the City of Edmonton, Alberta that would benefit from the intervention and therefore qualify for the study. All 10 schools invited agreed to participate in the intervention and research. We recruited cross-sectional samples of grade 5 students (age 10–11 years) for measurement, which included time-stamped pedometers, in the spring term from March to June in 2009 and 2011. This repeated cross-sectional design was chosen both to allow intervention effects to be assessed longitudinally at the school-level and to avoid the need for substantial imputation of missing data that would occur with a cohort design. Grade 5 students were of interest because most are pre-pubescent, and as a result boys and girls have similar body composition [18, 19], and have not experienced weight gain [20] or marked declines in physical activity [21–23] associated with puberty.

The comparison group was comprised of 20 schools, which were also located in Edmonton. These schools were drawn from a sample of randomly selected schools that participated in the 2008 “Raising healthy Eating and Active Living Kids” (REAL Kids) Alberta survey [24]. These schools had no prior involvement in health promotion. All 20 schools that were invited agreed to participate in the research. Grade 5 students were also recruited from these schools for measurement including time-stamped pedometers in the spring term from March to June in 2009 and 2011.

In 2008, to reach an effect size equivalent to a 20% increase in the consumption of vegetables and fruit in intervention schools, assuming 80% power with 5% type I error and considering the clustered nature of the data, a sample of 300 students from 10 intervention schools was estimated to be required. For the present study (2009 to 2011), where the primary outcome is pedometer-measured physical activity in step counts, we included two comparison schools for every intervention school as this provided 80% power to detect an intervention effect equivalent to a 15% increase in step counts. This calculation considered the clustered the clustered nature of the data, assumed a type I error rate of 5% and that 10 of the 20 comparison schools would drop out.

### Population

**Fig 1:**
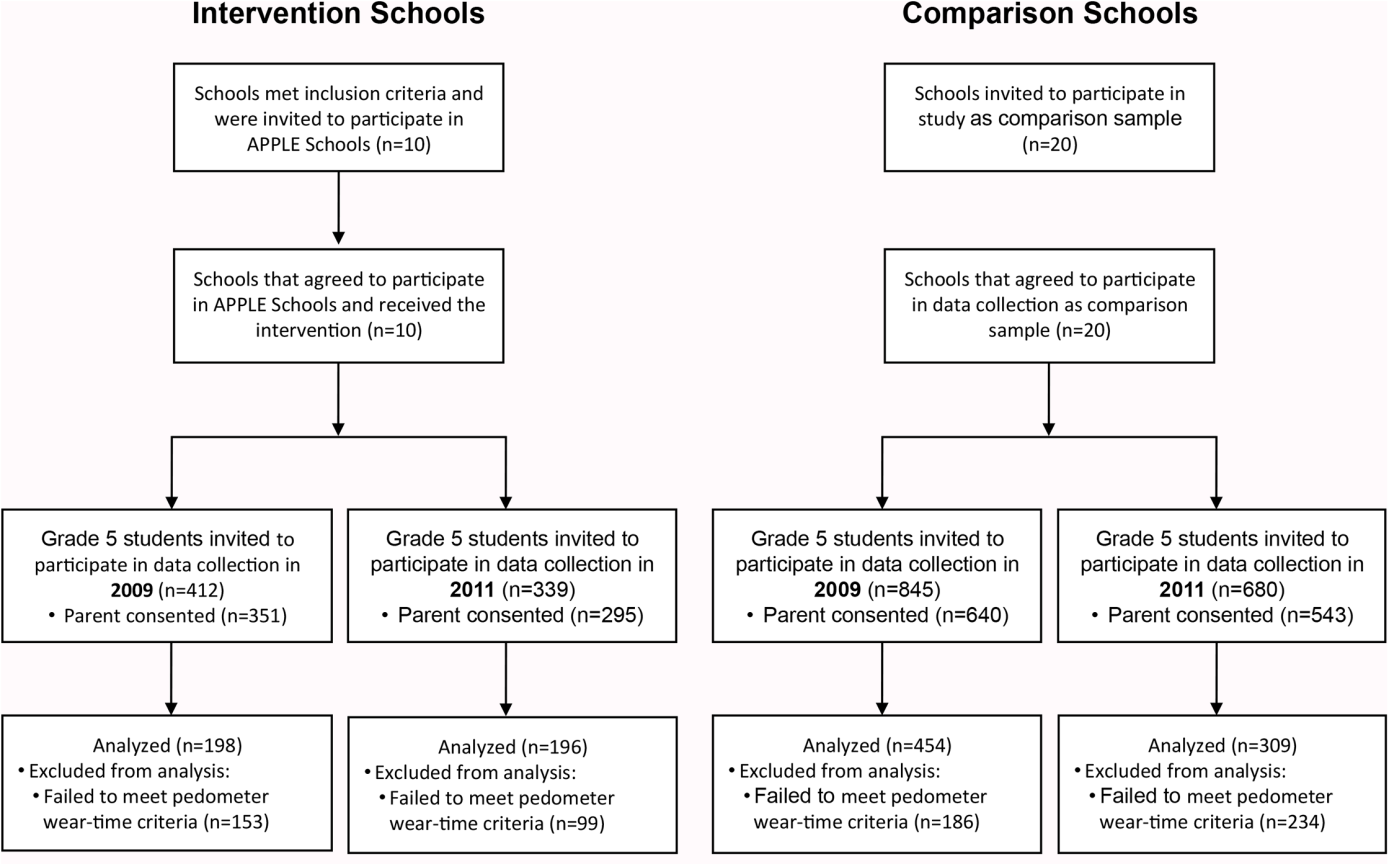
Description of enrollment of schools in the study and participation of grade 5 students in data collection, their pedometer usage, and analyses.

We invited all grade 5 students within each school to participate in the study. No students were excluded from participating. In 2009, among the 10 APPLE Schools, we provided all 412 students in grade 5 with home surveys and consent forms for their parents to complete and return to school. The home surveys collected demographic information including household income and parental educational attainment, as well as, information on children’s behaviours and parenting practices related to physical activity and nutrition (www.realkidsalberta.ca). A total of 358 parents completed these surveys (completion rate = 86.9%) and provided their consent for their child to participate in the evaluation. All students with parent consent assented to participate and completed student surveys on nutrition, physical activity, and other health related behaviours; 198 of these students also provided complete pedometer recordings and were included in analyses (completion rate = 48.1%). In 2011, only 339 students were enrolled in grade 5 within the APPLE Schools, though the survey completion rates and the number of complete pedometer recordings were similar (57.8%). In 2009 and 2011 respectively, we provided all 845 and 680 grade 5 students within the 20 comparison schools with home surveys and parent consent forms. Completion rates of the survey and pedometer recordings were similar in comparison schools in 2009 (53.7%) and 2011 (45.4%). The schools in our comparison sample also had fewer grade 5 students in 2011 than in 2009. A description of school enrollment in the study and participation of grade 5 students in data collection, their pedometer usage, and analyses is presented in Fig 1.

### APPLE Schools: the intervention

APPLE Schools is a school-based health promotion intervention based on the CSH approach, which aims to “make the healthy choice the easy choice”. CSH is described as, “an internationally recognized framework for supporting improvements in students’ educational outcomes while addressing school health in a planned, integrated, and holistic way” [25]. The framework is an integrated intervention approach that moves school health promotion beyond classroom-based education models and incorporates health promotion into the whole school environment. To realize CSH, actions must be addressed in four inter-related pillars, including: social and physical environments; teaching and learning; healthy school policy; and partnerships and services [25]. CSH is alternatively referred to as “Coordinated School Health” in the United States and as “Health Promoting Schools” in Australia and Europe [26].

A key component of APPLE Schools was the placement of a full-time school health facilitator in each school. Their role was to support the implementation of health promotion strategies in each school, to ensure that it met the schools’ unique needs for health promotion, and that it aligned with the core principles of CSH. The project’s goal was to create and sustain supportive physical and social environments that cultivate a healthy lifestyle through the collaboration of students, parents, teachers, and community stakeholders.

To reach children at high risk of physical inactivity APPLE Schools offered, on a daily basis, a variety of non-competitive, enjoyable activity choices both during and after school like weekly intramurals, dance, skipping and yoga clubs, walking initiatives, and playground programs. Additional steps taken to promote physical activity outside of school included improving access to after-school physical activity facilities and programs, organizing school-wide activities where students and parents collectively took part in promotions and events, and increasing traffic safety to promote and support active transportation. Monthly school newsletters where also distributed to parents describing affordable, easily accessible, and seasonally appropriate activities for children to participate in outside of school.

Comparison schools did not have access to a school health facilitator or to the health promotion materials used in APPLE Schools, though these schools received materials to implement Alberta Health’s provincial Healthy Weights Initiative. This initiative is an information and education campaign designed to support and encourage Albertans to lead healthier lifestyles (www.healthyalberta.com). Because the Healthy Weights initiative was implemented province-wide, all schools participating in the intervention also received these materials.

### Outcome of Interest: physical activity levels outside of school

We measured daily physical activity using the Omron Hj-720ITC time-stamped piezoelectric pedometer (Omron, Toronto Ontario, Canada). The accuracy and validity of the Omron pedometer has been demonstrated for children of this age [27], and under various conditions [28–31]. Pedometers were distributed to students in a classroom setting by trained evaluation assistants. Students were asked to wear their pedometers for nine consecutive days on the right hip directly in line with their right knee during all waking hours unless showering or swimming. Students also kept a log of their daily activities, including the duration of each activity and whether the pedometer was worn. On the third day of data collection, evaluation assistants returned to schools to encourage students to wear the pedometers and to complete their activity diaries. On the ninth day, research staff traveled to schools to collect pedometers and activity diaries, and download data to computers.

Pedometer records from the first and ninth days were not considered in data analysis due to varying administration and collection times. We defined a valid physical activity data file when the pedometer was worn for a minimum of eight hours [32]. In accordance with previous studies, only data from students with valid files on a minimum of two school days (Monday-Friday) and one non-school day (Saturday, Sunday, holidays) were included in analyses. When these criteria were met, pedometer-measured steps were complemented with step equivalents of non-ambulatory and non-wear time activities recorded in students’ activity diaries. In short, each activity recorded in the activity diaries was assigned a youth-specific metabolic equivalent task (MET) unit [33]. Based on the assigned METs, activities were categorized into intensity categories (i.e., light, moderate, moderate-to-vigorous, vigorous) [34, 35] and assigned a step per minute value [36]. Adult METs were used when youth specific values were not available [34]. We imputed information from the same hour(s) on other randomly selected valid days when students forgot to wear their pedometer and complete their activity diary. Steps were only imputed within an individual and within school days and weekend days. This method of imputation has been shown to replace data more accurately than traditional group-centered methods that replace missing data with the group mean [37]. These procedures are described in further detail elsewhere [7].

Students’ daily step-counts were averaged to represent school days and non-school days. Because active transportation to or from school is characteristic of behaviours on school days and school hours, we considered steps accumulated between 8:00am and 3:59pm (8 hours) Monday through Friday to be within school hours. We considered steps accumulated between 7:00am and 7:59am and 4:00pm and 8:59pm (6 hours) Monday through Friday to be within non-school hours. We calculated the average number of hourly steps during school hours and non-school hours by dividing the total steps accumulated during these periods by 8 and 6 hours respectively.

### Assessment of Other Covariates

Students’ gender was self-reported in the student survey. Evaluation assistants measured students’ standing height and body weight. Height was measured to the nearest 0.1 cm. Body weight was measured to the nearest 0.1 kg on calibrated digital scales. Students removed their shoes for both measurements. Body Mass Index (BMI) was calculated as weight divided by height^2^ (kg/m^2^). We defined overweight using the International Obesity Task Force BMI cut-off points adjusted to age and sex specific categories for children and youth [38]. Information on household income (≤$50,000; $50,001-$100,000; and >$100,000) and level of parental educational attainment (≤secondary, college, university or graduate school) were determined from parent responses in the home survey.

### Statistical Analysis

We used t-tests and chi-square tests to test for differences in daily and hourly step-counts and participant characteristics between students attending intervention and comparison schools in 2009 and 2011. We used multilevel linear regression methods to account for the clustering of students within schools, and analyzed subsets based on students’ body weight status, household income, and parental educational attainment. Analyses were adjusted for potential confounders including gender, and when applicable parental educational attainment and household income (see footnote in Tables 2 and 3). To examine the effect of the intervention on children’s physical activity we created an interaction term defined as the product of the binary variables year (0 = 2009, 1 = 2011) and intervention (0 = comparison schools, 1 = APPLE Schools). This term represents the two-year change among students attending APPLE Schools relative to the change among students attending comparison schools. Adjusted means, their differences and 95% confidence intervals (CI) were estimated from multilevel adjusted analyses and are presented in Tables 2 and 3. The relative difference in adjusted daily mean step-counts between children attending interventions schools and comparison schools in 2009 and 2011 was calculated from the absolute difference in the adjusted means in intervention and comparison schools divided by the adjusted mean steps per day in intervention schools. The relative change in steps between intervention and comparison schools from 2009 to 2011 was calculated by adding the relative difference in 2011 to the absolute value of the relative difference in 2009.

To generate cumulative distribution plots based on students’ step-counts on school days and weekend days and during school hours and non-school hours, students were stratified according to the evaluation year and intervention status. Next, students were ranked by step-counts from the lowest number of steps taken daily to the most, and their position in the distribution was plotted against their mean steps per day on school day and weekend days and against their mean steps per hour during school hours and non-school hours. These plots illustrate how the effects of the intervention are distributed across all students, and are complimentary to observed changes in average physical activity. We used STATA version 12 (StataCorp, College Station TX, USA) to perform the statistical analyses. The Health Research Ethics Board at the University of Alberta approved this study including data collection and informed parental consent forms. Written informed consent was obtained from a parent/guardian for every child who participated in the research and data collection.

## Results

Characteristics of grade 5 students from intervention and comparison schools in 2009 and 2011 are presented in Table 1. On average, students were 10.9 years old and 49.5% were girls. The proportions of boys and girls were comparable among intervention and comparison schools. One third of all students were overweight or obese, though the proportion was greater in intervention schools compared to comparison schools (36.9% vs. 31.0%; χ^2^ = 8.30 p<0.004). Approximately one quarter of all students came from low-income households and one third had parents with secondary school education or less. The proportion of youth from low-income and low-educated households was also higher in intervention schools than in comparison schools (household income < $50,000: 31.9% vs. 18.0% χ^2^ = 40.08, p<0.001; parental education ≤ secondary school: 29.2% vs. 24.1% χ^2^ = 5.44, p = 0.02 in intervention and comparison schools respectively; Table 1). Compared with students who provided valid pedometer data, those who did not were more likely to be boys (44.6% vs. 31.1%) and overweight (38.5% vs. 33%). Further, the failure to provide valid pedometer data was more common in 2011 compared with 2009 (39.7% vs. 34.2%).

**Table 1:** Characteristics of grade 5 students attending APPLE Schools and comparison schools in 2009 and 2011.

	2009			2011		
	APPLE Schools^a^n = 198	Comparison schools n = 454	p-value	APPLE Schools^a^ n = 196	Comparison schools n = 309	p-value
**Gender (%)**			0.28			0.60
Girls	47.2	50.8		51.0	49.1	
Boys	52.8	49.2		49.0	50.9	
**Weight Status (%)**			0.03			0.13
Overweight/obese	38.3	31.3		35.2	30.1	
Normal Weight	61.7	68.7		64.8	69.9	
**Household income (%)**						
<$50,000	34.7	18.1	<0.001	33.2	17.8	<0.001
$50,001–$100,000	40.0	37.2		31.2	31.8	
>$100,001	25.3	44.6		35.6	50.4	
**Parental Education (%)**			0.38			0.11
Secondary or less	31.9	27.9		26.0	19.8	
College	39.1	42.8		39.9	45.1	
University or graduate	29.0	29.3		34.1	35.1	

^a^: APPLE Schools = Alberta Project Promoting active Living and healthy Eating Schools

**Table 2:** Inequity in physical activity levels (steps/day) on school days (Monday-Friday) and weekend days (Saturday, Sunday, holidays) by grade 5 students attending APPLE Schools and comparison schools over a two-year interval (2009–2011) of a Comprehensive School Health intervention.

	2009					2011					Intervention Effect (a_2_–a_1_)—(c_2_–c_1_)	95% CI^a^	Relative Change^b^
	APPLE Schools (a_1_)	Comparison schools (c_1_)	Difference (a_1—_c_1_)	95% CI^a^	Relative Difference (a_1_-c_1_)/a_1_ x100	APPLE Schools(a_2_)	Comparison schools (c_2_)	Difference(a_1_–c_1_)	95% CI^a^	Relative Difference (a_2_-c_2_)/a_2_ x100			
**SCHOOL DAYS**													
**Overall** ^c^	11531	12699	-1168	-**1998;-338**	-10.1	13683	13630	53	-831; 936	+0.4	1221	**306;2135**	+10.5
**Weight Status** ^c^													
Overweight/obese	10803	12449	-1646	**-2798;-493**	-15.2	13169	13580	-411	-920;1741	-3.1	1235	-372;2842	+12.1
Normal weight	11926	12831	-905	-1873;62	-7.6	13929	13614	315	-710;1341	+2.3	1221	**92;2350**	+9.9
**Income** ^d^													
<$50,000	11371	12729	-1358	-3203;488	-11.9	13646	12535	1111	-1111;3335	+8.1	2469	**42;4897**	+20.1
$50,001-$100,000	11335	12463	-1128	-2314;58	-10.0	13159	13658	-499	-1947;949	-3.8	629	-1243;2500	+6.2
>$100,000	11439	13046	-1607	**-3113;-102**	-14.1	13482	13537	-55	-1470;1359	-0.4	1552	-315;3420	+13.6
**Education** ^d^													
<Secondary	10953	12763	-1810	**-3312;-307**	-16.5	13759	13169	590	-1135;2316	+4.3	2400	**515;4285**	+20.8
College	11524	12448	-924	-2080;231	-8.0	13400	14235	-835	-2023;354	-6.2	90	-1388;1568	+1.8
University/graduate	11463	13051	-1588	2816;-360	-13.9	13618	13347	271	-1070;1612	+2.0	1859	**285;3433**	+15.8
**WEEKEND DAYS**													
**Overall** ^c^	9212	11186	-1974	**-2938;-1009**	-21.4	12148	12121	27	-1004;1059	+0.2	2001	**600; 3402**	+21.6
**Weight Status** ^c^													
Overweight/obese	8935	10638	-1703	**-3282;-126**	-19.1	11404	10954	450	-1375;2275	+3.9	2153	-247;4553	+23.0
Normal weight	9336	11332	-1996	**-3203;-790**	-21.4	12792	12540	252	-1011;1515	+2.0	2248	**516;3980**	+23.4
**Income** ^d^													
<$50,000	8510	10140	-1630	-3967;708	-19.2	12002	9997	2005	-936;4945	+16.7	3635	**136;7133**	+35.9
$50,001-$100,000	9366	11288	-1922	-3893;49	-20.5	12229	12929	-700	-3089;1688	-5.7	1221	-1652;4094	+14.8
>$100,000	10039	12474	-2435	**-4764;-107**	-24.3	11020	12256	-1236	-3361;887	-11.2	1199	-1952;4349	+13.0
**Education** ^d^													
<Secondary	7796	10644	-2848	**-4546;-1150**	-36.5	12139	11658	481	-1546;2509	+4.0	3329	**694;5965**	+40.5
College	9986	11573	-1587	-3240;67	-15.9	10778	12857	-2079	**-3802;-356**	-19.3	-493	-2880;1894	-3.4
University/graduate	9059	11189	-2129	**-3872;-387**	-23.5	13555	11725	1829	-63;3721	+13.5	3959	**1478;6439**	+37.0

^a^; CI = Confidence interval

^b^: |relative difference in 2009|+Relative difference in 2011

^c^:adjustments for potential confounders included: gender, parental education, and household income

^d^: adjustments for potential confounders included: gender

Note: bolded 95% CI indicate statistical significance at p<0.05.

**Table 3:** Inequity in physical activity levels (steps/hour) during school (8:00am–3:59pm) and non-school hours (7:00–7:59am & 4:00–8:59pm) by grade 5 students attending APPLE Schools and comparison schools over a two-year interval (2009–2011) of a Comprehensive School Health intervention.

	2009					2011					Intervention Effect (a_2_–a_1_)—(c_2_–c_1_)	95% CI^a^	Relative Change^b^
	APPLE Schools (a_1_)	Comparison schools (c_1_)	Difference (a_1—_c_1_)	95% CI^a^	Relative Difference (a_1_-c_1_)/a_1_ x100	APPLE Schools(a_2_)	Comparison schools (c_2_)	Difference (a_1_–c_1_)	95% CI^a^	Relative Difference (a_2_-c_2_)/a_2_ x100			
**SCHOOL HOURS**													
**Overall** ^c^	860	946	-86	**-150;-23**	-10.0	947	986	-39	-105;27	-4.1	47	-12;107	+5.9
**Weight Status** ^c^													
Overweight/obese	827	941	-114	**-219;-9**	-13.8	938	1013	-75	-190;40	-8.0	39	-69;147	+5.8
Normal weight	887	951	-64	-131;1	-7.3	956	981	-25	-95;45	-2.6	40	-33;112	+4.7
**Income** ^d^													
<$50,000	856	988	-132	-265;0	-15.5	962	1012	-50	-208;108	-5.2	83	-85;251	+10.3
$50,001-$100,000	827	913	-86	**-161;-12**	-10.5	919	972	-53	-144;38	-5.8	33	-82;149	+4.7
>$100,000	869	952	-83	-181;13	-9.7	963	964	-1	-91;91	0.0	84	-38;206	+9.6
**Education** ^d^													
<Secondary	862	938	-76	-176;24	-8.8	985	921	64	-50;178	+6.5	140	18;262	+15.3
College	855	940	-85	-184;14	-9.9	921	1030	-109	**-210;-9**	-11.9	-25	-120;71	-1.9
University/graduate	847	963	-115	**-202;-29**	-13.6	937	981	-44	-138;50	-4.7	72	-30;173	+8.9
**NON-SCHOOL HOURS**													
**Overall** ^c^	778	856	-78	-166;9	-10.1	1016	958	58	-36;152	+5.7	137	**31;242**	+15.8
**Weight Status** ^c^													
Overweight/obese	725	828	-103	-218;12	-14.2	945	936	9	-123;143	+1.0	113	-62;287	+15.2
Normal weight	806	871	-65	-171;41	-8.1	1046	963	83	-30;195	+7.9	148	**15;281**	16.0
**Income** ^d^													
<$50,000	753	805	-52	-242;138	-6.9	990	744	246	**17;476**	+24.9	298	**46;550**	+31.8
$50,001-$100,000	787	864	-77	-225;72	-9.7	956	982	-26	-207;155	-2.7	50	-171;272	+7.0
>$100,000	746	907	-161	-329;7	-21.6	958	975	-17	-174;140	-1.8	144	-70;358	+19.8
**Education** ^d^													
<Secondary	681	875	-194	**-354;-35**	-28.6	972	963	9	-178;196	+0.9	203	-13;420	+29.5
College	778	831	-53	-176;72	-6.7	998	1019	-21	-149;108	-2.1	32	-140;203	+4.7
University/graduate	788	896	-108	-235;18	-13.8	1033	920	113	-24;250	+10.9	221	**40;403**	+24.7

^a^; CI = Confidence interval

^b^: |relative difference in 2009|+Relative difference in 2011

^c^:adjustments for potential confounders included: gender, parental education, and household income

^d^: adjustments for potential confounders included: gender

Note: bolded 95% CI indicate statistical significance at p<0.05.

**Fig 2:**
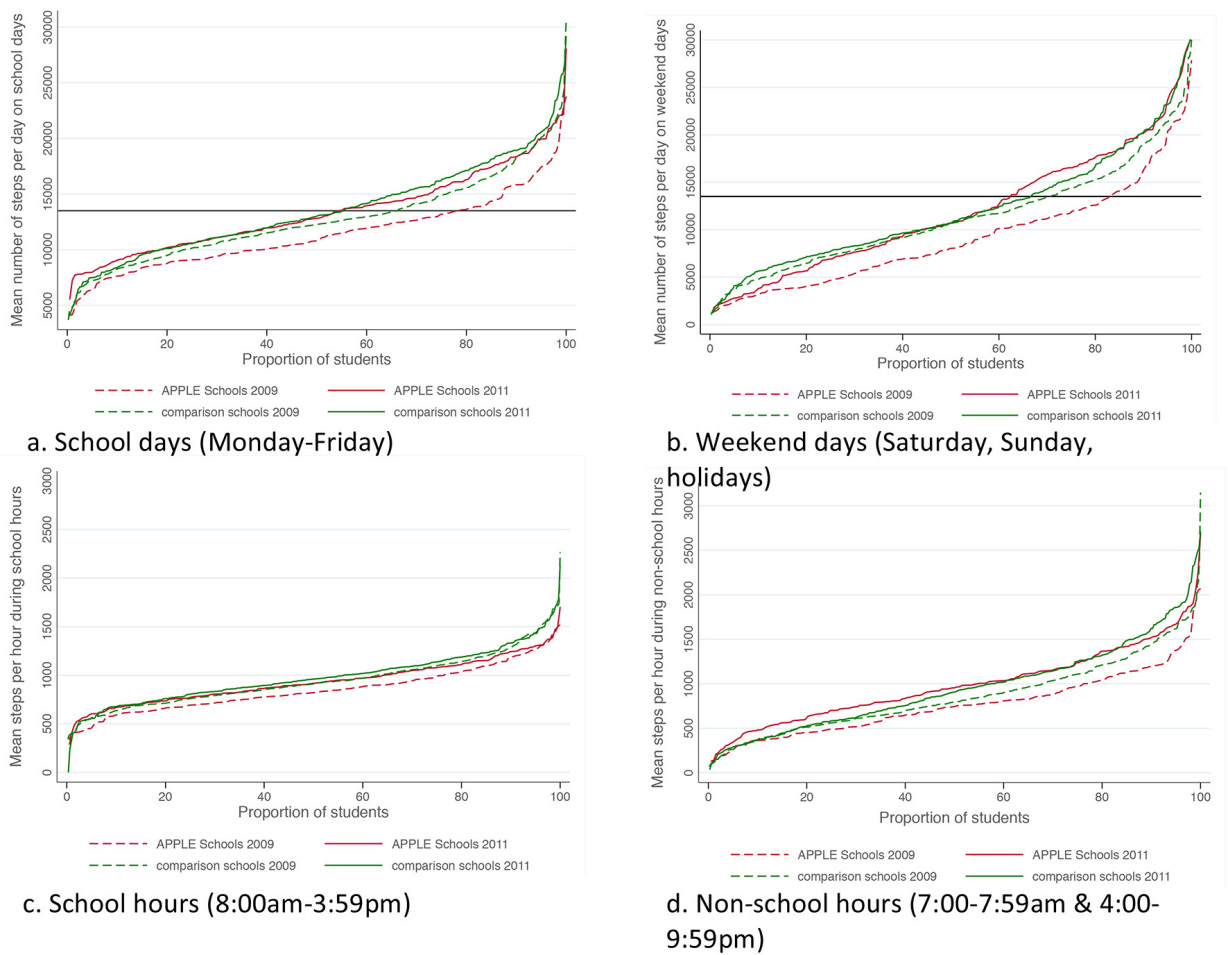
Mean number of steps taken among all students from APPLE Schools and comparison schools in 2009 and 2011 on a) school days; b) weekend days; c) school hours; and d) non-school hours.

In 2009, children took approximately 2000 fewer steps per day on weekend days than on school days (10,555 vs. 12,311 steps/day; 95% CI = 1381; 2131) equating to a 14.3% difference in daily steps taken on weekend days. Children also took approximately 100 fewer steps per hour outside of school hours than during school hours (831 vs. 915 steps/hour; 95% CI = 54; 115). Across all time periods in 2009, children from intervention schools took fewer steps per day and fewer steps per hour than those from comparison schools (Fig 2A–2D). The discrepancy in activity levels between children from intervention and comparison schools was most pronounced outside of school, particularly on weekend days with children from intervention schools achieving 21.4% fewer steps daily than children from comparison schools (9212 vs. 11186 steps/day; 95% CI = -2938; -1009; Table 2).

From 2009 to 2011, physical activity levels increased in both intervention and comparison schools, though the increases were significantly greater among children from intervention schools (Fig 2A–2D). Specifically, estimates from multilevel analyses adjusted for potential confounders showed that daily step-counts increased 10.5% more on school days (+1221 steps/day; 95% CI: 306; 2135) and 21.6% more on weekend days (+2001 steps/day; 95% CI: 600; 3402) in children from intervention schools than in children from comparison schools (Table 2). Further, hourly steps-counts increased 15.8% more during non-school hours (+137 steps/hour; 95% CI: 31; 242) in children from intervention schools than in children from comparison schools (Table 3).

### Subgroup analyses: Income and Education

In 2009, children in all income and education groups from intervention schools were less active relative to those from comparison schools (Fig 2). The inequity in physical activity between intervention and comparison schools was more pronounced on weekend days. This was particularly evident among children whose parents had the lowest educational attainment. These children took 16.5% fewer steps per day than those from comparison schools on school days (-1810 steps/day; 95% CI: -3312; -307, Table 2) and 36.5% fewer steps per day on weekend days (-3164 steps/day; 95% CI: -4546; -1150, Table 2). This group of children also experienced the greatest relative difference in physical activity from school hours to non-school hours (-8.8% to -28.6%, respectively, Table 3).

The APPLE schools intervention led to significantly greater increases in daily step counts among children within the lowest income and education groups from intervention schools relative to children within these same groups from comparison schools on school days (<$50,000: effect size = 2400; 95% CI: 515; 4285; <secondary school education: effect size = 2469; 95% CI: 42; 4897, Table 2), and weekend days ((<$50,000: effect size = 3635; 95%CI: 136; 7133; education effect size = 3329; 95%CI: 694; 5965, Table 2). Similar effects were observed during and outside school hours among children within these groups from intervention schools. These increases were more pronounced than those observed among children within the middle and high parental education and within the middle and high income groups (Tables 2 and 3).

### Subgroup analyses: Overweight

In 2009, on school days, overweight children from intervention schools took 15.2% fewer steps per day than overweight children from comparison schools (-1646 steps/day; 95% CI: -2798; -493, Table 2). On weekend days, the discrepancy in activity levels between intervention and comparison schools was more pronounced. Specifically, overweight children from intervention schools took 19% fewer steps (-1703 steps/day; 95% CI: -3282; -126, Table 2) than overweight children from comparison schools. In 2009, overweight children from intervention schools also took 13.8% (-114 steps/hour; 95% CI: -219; -9, Table 3) and 14.2% (-103 steps/hour; 95% CI: -218; 12, Table 3) fewer steps per hour during school and non-school hours, respectively.

Following the intervention, daily step counts among overweight children increased approximately 12.1% more on school days (effect size = 1235; 95% CI: -372; 2842) and 23.0% (effect size = 2153; 95% CI: -247; 4553) more on weekend days in intervention schools than in comparison schools, though these effects were not statistically significant. Findings were similar during school hours and non-school hours (Table 3).

## Discussion

The data presented here provide novel evidence suggesting that school-based health promotion activities translate into active lifestyle behaviours after school hours and reduce physical activity related inequalities among children. First, we showed that inequities are more pronounced when children are outside of school time. Second, we showed that after two years of CSH programming physical activity levels of overweight and socioeconomically disadvantaged students on weekend days and outside of school hours increased to the same extent or greater than their normal weight or more advantaged peers. Overall, “exposure” to CSH, like the APPLE Schools program, not only normalized physical activity levels of children living in disadvantaged neighbourhoods, but it also effectively reached these children during the windows of time when their activity levels were lowest.

Several studies have provided evidence of the disparity in habitual physical activity levels between children of various weight and socioeconomic status groups [39]. Yet none, to our knowledge, have demonstrated whether these disparities persist on all days of the week. The data presented here demonstrate that inequities in physical activity are exacerbated on weekend days and outside of school hours. Further, they confirm our hypothesis that children at greater risk of inactivity may need additional support to be active outside of the school setting. It is possible that without structured, enjoyable, and safe opportunities for physical activity, overweight and socioeconomically disadvantaged children engage in more sedentary activities and are less likely to pursue alternative ways to be active during their discretional time than their normal weight and more advantaged peers. Future policies and health promotion programs should consider alternative ways to engage these children in physical activities outside of school to reduce the inequities experienced by these children during this period.

CSH appears to be an effective way to reduce physical activity inequities among children. Recently, we showed that the APPLE Schools program is an efficacious approach for increasing weekly average physical activity levels of children living in socioeconomically disadvantaged neighbourhoods and disadvantaged subgroups, including overweight and low socioeconomic status children [40]. Results from the present study further extend this argument by demonstrating that though the program was implemented during school hours, CSH may also effectively reduce physical activity inequalities outside of the school setting. Perhaps the effects on physical activity observed outside of school were realized because the program used a multifaceted approach. This approach is an integral part of CSH that facilitates behaviour change by engaging key stakeholders including students, parents, school staff, and community members. Reviews demonstrate that incorporating family and community members enhances the effectiveness of interventions delivered at school [41–46]. These stakeholders likely assist in establishing a support-network for children beyond the school setting, which encourages them to be active at home and in their community. This network is critical to improving physical activity levels outside of school, particularly among those at risk of low physical activity, as home and community environments are often full of barriers to physical activity. To engage and involve parents in the APPLE Schools program, parents were invited to sit on planning and review committees like the “APPLE Core committee”, newsletter were sent home regularly with information about affordable opportunities for physical activity in their community, and parents were asked to volunteer to at school events. Community stakeholders were also invited to sit on school committees. They also helped to improve access to and affordability of recreational facilities in school neighbourhoods. The data presented here strengthen the evidence that incorporating stakeholders outside of school enhances the effectiveness of interventions delivered at school.

Strengths of the present study include the use of an objective measure of physical activity, the large sample size, adjustments for non-ambulatory and non-wear time activities, and height and weight measurements to assess body weight status. There are a few limitations, however, that should be addressed. First, schools were not randomized to an intervention or control condition. As a result, the risk of selection bias may be increased and the effect size associated with the intervention may be inflated, and the generalizability of results limited. Second, schools, rather than students, were followed longitudinally. Therefore, results must be interpreted at the school level rather than the individual level. Third, low compliance rates with pedometer inclusion criteria were low. It is unlikely that this influenced the size of the observed effect, however, as these rates were similar among intervention and comparison schools. Additionally, when parent and student consent and survey completion rates are considered, the participation rate improved considerably such that they approximated 80–85%. Third, parent responses and student records in activity diaries also remain subjective and prone to bias. However, previously we showed that adjustments to pedometer-measured steps for step-equivalents estimated from activity diaries are relatively even across activity groups [7]. Accordingly, it is unlikely that this affected the observed effect size.

## Conclusion

The present study demonstrated that physical activity inequalities are exacerbated outside of the school setting. However, exposure to multi-faceted school-based health promotion programs may reduce physical activity inequalities for overweight/obese and socioeconomically deprived children outside of school.

## Supporting Information

S1 TREND Checklist Trend checklist of information to include when reporting a randomized trial. (DOCX)

S1 Protocol Study protocol for The Alberta Project Promoting active Living and healthy Eating in Schools (APPLE Schools) approved by the Health Ethics Research Board at the University of Alberta. (PDF)
