# Supplementary material for: Does School-Based Health Promotion Affect Physical Activity on Weekends? And, Does It Reach Those Students Most in Need of Health Promotion?
Source: PLoS One. 2015 Oct 21;10(10):e0137987. doi: 10.1371/journal.pone.0137987 (PMC4619216; doi:10.1371/journal.pone.0137987)
Supplement: S1 Protocol — (PDF) [file pone.0137987.s002.pdf]

S2. Study protocol for The Alberta Project Promoting active Living and healthy Eating in Schools (APPLE Schools) approved by the Health Ethics Research Board at the University of Alberta.

The proposed activities consist of 1) the intervention; and 2) evaluation research.

1) The Intervention "The Alberta Project Promoting active Living and healthy Eating in Schools (APPLE Schools)": We will hire health promoters/educators, one for each school, who will be positioned in the school far a 4-year period to develop, implement and execute the intervention. This person's first responsibility will be to customize the intervention to the specifics of the schools. This will be a process that includes involvement of school staff, parents and students. In various formats the intervention is likely to include the following:

- *A curriculum for all students that stresses the importance of good nutrition and physical activity.*
- *A school policy on food and nutrition, consistent with the original Annapolis Valley Health Promoting Schools project, which includes,*
  - *Healthy menu and food offerings;*
  - *Cost of food to help ensure affordability for all students; and*
  - *Training of food service personnel.*
- *A school environment that is committed to healthy nutrition: existing lunch contracts with fast food providers will not be renewed; soft drink-sales will be halted; vending machines will support only healthy food choices.*
- *All students will have access to daily physical activity.*
- *Improves access to after-school physical activity facilities and programs for students.*
- *Each school community will be asked to develop as community-based plan to involve and educate parents, staff, and community groups, volunteers and organizations.*
- *Improved traffic safety so that students are more likely to walk and cycle to school.*

2) The Alberta Project Promoting active Living and healthy Eating in Schools (APPLE Schools) promotes "Make the Healthy Choice the Easy Choice". Raising healthy Eating Active Living kids in Alberta (REAL Kids Alberta) is used for the quantitative evaluation of APPLE Schools. REAL Kids Alberta will evaluate APPLE Schools in order to see if APPLE Schools project is marking a difference in improving dietary intake and increasing physical activity. The findings from the evaluation will provide valuable information to help inform and enhance APPLE Schools implementation.
